# Primary intracardiac leiomyoma: rare case report and literature review

**DOI:** 10.1186/s13019-024-03083-1

**Published:** 2024-09-28

**Authors:** Haneen Al-Maghrabi, Uthman Aluthman, Ahmed Jamjoom, Ibrahim Zabani, Jaudah Al-Maghrabi

**Affiliations:** 1 Department of Pathology and Laboratory Medicine, King Faisal Specialist Hospital and Research Center, P.O. BOX 80205, 21589 Jeddah, Saudi Arabia; 2 Department of Cardiovascular, Cardiac Surgery Section, King Faisal Specialist Hospital and Research Center, Jeddah, Saudi Arabia; 3 Department of Anesthesia, King Faisal Specialist Hospital and Research Center, Jeddah, Saudi Arabia; 4 Department of Pathology, Faculty of Medicine, King Abdulaziz University, Jeddah, Saudi Arabia

**Keywords:** Heart, Cardiac, Leiomyoma, Left ventricle

## Abstract

Benign cardiac neoplasms are relatively uncommon. Cardiac leiomyomas are usually diagnosed as a benign metastasizing leiomyoma or as a part of intravenous leiomyomatosis spectrum. Primary cardiac leiomyomas are extremely rare and should be diagnosed after ruling out the involvement of systemic leiomyomas. Only nine cases were found in the literature that described De novo occurrence of primary intra-cardiac leiomyoma. In this study, we present a case of 60-year-old female patient with a large pedunculated mass located in the left ventricle. Histopathology examination and immunohistochemistry aid confirmed the diagnosis of benign leiomyoma. No evidence of extra cardiac lesions was detected in the patient. The patient remained healthy with no signs of recurrence four years after the surgical resection. Benign cardiac tumors are not often seen, but when they do occur, they can present a serious risk to life. This is particularly significant because these tumors can detach and cause embolization, leading to the development of strokes. Moreover, these individuals do not show any clinical symptoms, making their detection quite challenging. When there is a suspicion, it is advised to utilize echocardiography and other imaging techniques to verify the presence of a tumor. In this report, we present a rare case and provide differential diagnoses, along with a review of the literature.

## Introduction

De novo occurrence of primary intra-cardiac neoplasms is uncommon. A study series reported an incidence of only 0.001 − 0.03% of primary cardiac neoplasms detected in autopsies [1]. The 5th edition of World Health Organization (WHO) classification system of benign tumors and tumor-like lesions of the heart does not include benign primary cardiac leiomyoma [2]. Approximately 75% of primary cardiac tumors are believed to be benign. A greater occurrence has been reported, mainly observed on the left side, particularly in the left atrium. Cardiac myxoma is considered the most common benign cardiac neoplasm, representing an estimated incidence of one per million per year and accounting for 50–75% of all benign tumors [3]. Rhabdomyomas represent common primary cardiac neoplasms in children and young adults [4]. Other benign cardiac tumors include lipoma, papillary fibroelastoma, hemangioma, and neurofibroma; while primary cardiac leiomyomas are extremely rare. Only nine case reports found in a review of the English literature (Table 1). Patients with metastatic benign leiomyoma and those with venous seeding were not included in the study. Clinical and radiological differential diagnoses vary widely for primary cardiac intraventricular mass. However, histopathology examination along with immunohistochemistry stains is needed to confirm the final diagnosis. In this study, we describe a case of a primary left ventricular mass in a 60-year-old lady, who proved clinically to have no other leiomyoma elsewhere. Further analysis of this neoplasm, including possible pathophysiology, histology and differential diagnosis are discussed below.

**Table 1:** Summary of reported cases of primary Cardiac Leiomyomas (n=10)

	Author [Reference]	Age/ Sex	Hospital Investigation	Presentation	Size (cm)	Site	Number of lesions	Follow-up
**1**	Horton et al. (2006) [13]	31 year/ Female	CT: Large pericardial effusion, ill-defined pericardial mass	Epigastric and lower thorax pain	10 × 8 × 3.5	Posterior wall of the left atrium	Solitary	43 month- asymptomatic No R/M
**2**	Qin et al. (2010) [14]	13 year/ Male	Echo: Extended into the cardiac chamber	Incidental detection	6.5 × 5.5 × 4	Lateral wall of right ventricle	Solitary	1 year-asymptomatic No R/M
**3**	Melo et al. (2012) [9]	7months/ Male	MRI: mass occupying most of the right ventricle, into the ventricles outflow tracts	Incidental detection	3.5 × 3.5	Right ventricle	Solitary	1 month- asymptomatic No R/M
**4**	Song et al. (2014) [6]	24 year/ Female	CT scan: close to right coronary sinus into right chamber, significant right ventricular outflow obstruction	Incidental detection	2.4 × 2.8	Right ventricle	Solitary	4 months- asymptomatic No R/M
**5**	Li et al.(2016) [15]	47 year/ Female	Echo: rodlike mass in the right ventricle extending into right pulmonary artery. CT: confirmed Echo results	Cough, cardiac mass on CT scan	10.0 × 4.0 × 3.5	Right ventricle	Multiple	30 months-asymptomatic No R/M
**6**	Gaur et al. (2017) [5]	14 year/ Male	Echo: uniform consistency mass, prolapsing into right atrium. Attached to atrioventricular septum	Shortness of breath, palpitation	6.2 × 6.5 × 4.0	Right ventricle	Solitary	6 months- asymptomatic No R/M
**7**	Careddu et al. (2017) [16]	43 year/ Female	Echo: severe right ventricular outflow tract obstruction and tricuspid valve regurgitation	Dyspnea, palpitation, and syncope	5.5 × 3.2 × 2.3 (largest mass)	Right ventricle	Two masses	14 years- asymptomatic No R/M
**8**	Ni et al. (2017) [17]	38 year/ Male	CT: mass between aortic root and left atrial roof, compressed left atrium&left superior pulmonary vein	Palpitation, recurrent presyncope, sweating & nausea	3 × 3.8	Intrapericardial, Roof of the left atrium	Solitary	4 years- asymptomatic No R/M
**9**	Yahaya J (2020) [18]	74 year/ Male	Discovered during autopsy	Sudden death/ no known previous history of heart disease	4 × 3 × 2	lower left ventricle extending to left ventricular septum	Solitary	Died suddenly and unexpectedly
**10**	The present case	60 year/ Female	CT: lobulated well-defined hypodense mass MRI: confirmed CT results	Incidental detection	6 × 4.5 × 2.5	Left ventricle	Solitary	4 years- asymptomatic No R/M

*NA: Not available, *R: Recurrence, *M: Metastasis, *Echo: Echocardiogram, *CT: Computed tomography, *MRI: Magnetic resonance imaging.

## Case presentation

This case study describes a 60-year-old female, smoker, with a history of permanent pacemaker (PPM) implantation 8 years prior to presentation. She presented after undergoing a routine checkup as a part of her work policy which showed evidence of atrial fibrillation (A-fib) on electrocardiography (ECG). The patient described a history of intermittent dizziness, which spiked and self-terminated. She had a history of PPM implantation done 8 years ago which was done for her after she complained of dizziness and a holter monitor done for her which showed significant pauses. Pacemaker switched off two years ago after implantation when her checkup showed that the patient rarely used in past years. She had a longstanding smoking history. No history of cardiac illness or any other malignancies in her family. On physical examination, the patient was vitally stable, afebrile. Blood pressure within normal limits. Heart rate was 77 beats per minute, temperature 36 degrees Celsius, respiratory rate of 20 beats per minute, oxygen saturation 96% on room air, blood pressure 116/86 mmHg. ECG showed episodes of ventricular tachycardia.

**Fig. 1:**
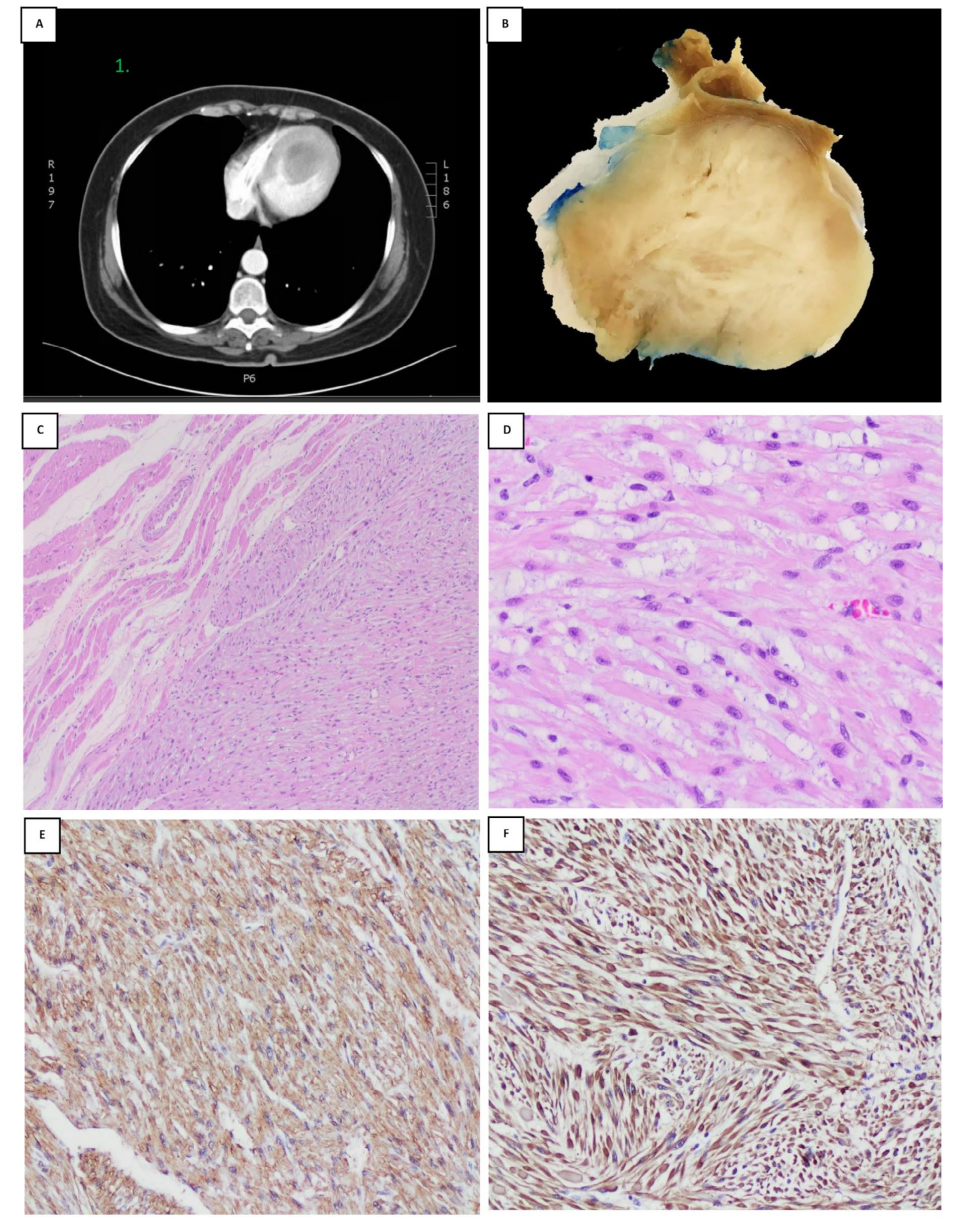
Radiology, representative hematoxylin and eosin (H&E) and immunohistochemical staining of cardiac leiomyoma. **(A)**: Computed Tomography (CT) scan with contrast of the chest revealed lobulated well-defined hypodense mass occupying the left ventricle measures 5 cm. no evidence of intrathoracic metastasis. **(B)**: Gross examination of a well circumscribed mass containing white-tan whorled cut surface. No areas of necrosis are seen. **(C)**: Sharply demarcated neoplastic cells composed of smooth muscle bundles proliferation, arranged in whorled pattern. No mitosis, necrosis, nor atypia seen (H&E; 4x). **(D)**: Tumor cells are composed of spindle cells with classical cigar-shaped nuclei, interlacing fascicles (H&E; 20x). **(E)**: Tumor cells expressing strong SMA staining (10x). **(F)**: Tumor cells expressing strong H-caldesmon staining (10x)

Her present echocardiogram showed a large pedunculated mass, not mobile, oval shape with smooth margins, attached to the apex and occupying most of the left ventricle cavity. The mass is obliterating the left ventricular outflow tract (LVOT) and interfere with mitral valve function. Sever left ventricular systolic dysfunction (LVSD) noted with left ventricular ejection fraction (LVEF) 20%. The left atrium and right atrium appeared of normal size and function, with intact interatrial septum and no evidence for an arterial septal defect. The mitral valve was normal in structure and function with no evidence of stenosis or prolapse. All other valves are normal in function and structure. No evidence of pericardial effusion. The right ventricle was normal in size and function, normal wall thickening, and normal right ventricular systolic function. Cardiac catheterization showed normal coronary arteries. A computed tomography (CT) scan revealed a lobulated well-defined hypodense mass occupying the left ventricle, measuring 5 × 4.3 × 4 cm (Fig. 1A). Cardiac magnetic resonance imaging (MRI) confirmed the presence a left ventricular mass. No pericardial or pleural effusion, consolidation, pulmonary nodules. No evidence of intrathoracic or intraabdominal masses found, and normal cerebral vasculature noted. The patient admitted for left ventricular mass excision through ventriculotomy. The patient brought to the operating room, intubated under general anesthesia and lined up. She prepped and draped in supine position with sterile fashion. Median sternotomy with pericardiotomy done. Aorta and right atrial cannulation done. The patient was on cardiopulmonary bypass. Vent and cardioplegia cannula were inserted in the aorta and aorta was crossclamped and cardioplegia delivered. Through left ventriculotomy incision, the mass was adherent to the ventricular wall and was easily dissected with left ventricular incision repair. Frozen section consultation showed a spindle cell tumor favoring muscular origin. Complete surgical resection was performed with no complications. The patient tolerated the procedure well and was transferred to cardiac surgery intensive care unit (ICU) in stable condition with good hemodynamics. The specimen was sent for histopathology analysis. Gross pathological examination of the specimen revealed a firm, oval, well-circumscribed mass with a smooth outer surface, measuring 6 × 4.5 × 2.5 cm (Fig. 1B). Histopathology examination revealed well-delineated benign neoplastic tissue composed of interlacing bundles of smooth muscle cells separated by a small amount of connective tissue (Fig. 1C). These cells were elongated cigar-shaped spindle cells with fibrillary acidophilic cytoplasm, characteristic of leiomyoma (Fig. 1D). There was a small focal area of degenerative nuclear atypia changes. However, there was no evidence of increased mitotic activity [less than 1/ 10 high power field (HPF)]. No evidence of necrosis or hyper cellularity were seen. Tumor cells were positive for desmin, smooth muscle actin (SMA) (Fig. 1E), muscle-specific antigen (MSA), and H-Caldesmon (Fig. 1F). Estrogen (ER) and progesterone receptors (PR) showed faint nuclear staining. They were negative for myogenin and myoglobin. Overall, the patient’s histology and immunohistochemistry results were diagnostic of leiomyoma.

Postoperative echocardiogram showed left ventricular systolic function moderate to severe impairment with ejection fraction of 30 to 35. No evidence of left ventricular mass. No pericardial effusion. Her laboratory investigation of White Blood Count (WBC) 11.7, Hemoglobin 13.1, Platelet 333, Sodium 143, Potassium 4.6, Urea 7.4, Creatinine 83, Estimated glomerular filtration rate (EGFR) more than 60. On physical examination, chest is clear on auscultation bilateral, no added sound. Cardiovascular (CVS) examination, S1, S2 plus 0. Abdomen was soft and lax, nontender, no organomegaly. Extremity good peripheral pulses. No lower limb edema. Central nervous system (CNS) examination sensory and motor examination grossly intact. Patient followed up in the outpatient clinic, her wound was clean, and she looked healthy. Four-year follow up, the patient is healthy and doing well with no disease recurrence.

## Disscussion

Leiomyomas are benign smooth muscle neoplasms that are commonly diagnosed in the female genital system, particularly the uterus. Extra-uterine leiomyomas are rarely diagnosed and are a greater diagnostic challenge. The most common extra-uterine leiomyomas occur in the genitourinary system, gastrointestinal tract, skin, and soft tissue. Interestingly, leiomyomas with unusual growth patterns can be seen, such as benign metastasizing leiomyomas [5]. Primary intra-cardiac leiomyomas are rarely seen and are typically part of benign metastasizing leiomyoma or intravenous leiomyomatosis spectrum [6]. Benign metastasizing leiomyoma is characterized by the presence of pelvic or uterine leiomyoma along with histologically identical neoplasm of smooth muscle proliferation outside the uterus and pelvis, which is commonly involved in the lung and then the heart. In cases of benign metastasizing leiomyomas, there is no evidence of vascular involvement as seen in intravenous leiomyomatosis [7]. The latter is a benign smooth muscle proliferation that occurs as lymphatic or venous intraluminal growth and resides in lymphatic channels located in the uterine parametrium, sometimes in the absence of primary uterine myoma. They can spread in the target channels up to the inferior vena cava and eventually to the right side of the heart [8]. In the present case, all clinical and imaging results ruled out the presence of any systemic, extra cardiac myomas, which confirmed the diagnosis of primary cardiac origin. An English literature review (up to February 2024) revealed only a limited number of published case reports that confirmed primary intra-cardiac leiomyoma. These reported cases showed a slight male predominance, with a mean age of 19.7 years. Four out of nine cases were detected incidentally due to other medical causes clinical work-up, and one of them during autopsy (Table 1). Despite the variable tumor sizes detected, the mean size grossly measured 5.9 cm. The patient we have presented was asymptomatic, and her diagnosis was made because she went in for a routine check-up. Interestingly, most cases (6 out of 9) showed the lesion in the right ventricle, which may be correlated to the tumor embryogenesis and tumor pathogenesis. Previous studies have suggested that primary intra-cardiac leiomyoma is related to intra myocardial blood vessels tunica media [6, 9]. In the present case, although we did conduct extensive sampling, the origin of the tumor was uncertain. However, some embryologic theories suggested that smooth muscle neoplasm can originate primarily from cardiac myocytes. In cardiac embryogenesis, cardiac outflow tract is introduced ultimately to primitive heart tube formation (PHT) [10]. The initial increase in cardiac mesodermal cells can cause the formation of primary heart tube through first heart field. The second heart field (SHF) is considered a rich active cellular progenitor that contributes actively to both arterial and venous elongation of the PHT [11]. It is well known that the fetal signaling defect degenerated in the SHF can lead to congenital heart disease [12]. Regarding the fact that all previously reported cases were presented with a right ventricular mass, the SHF anterior cellular portion contributed to the cardiac ventricular outflow. Moreover, anterior SHF progenitor cells that are positive for multipotent Islet-1 (Isl-1) are more capable for smooth muscle differentiation [11]. Although our present data do not provide the molecular tumor origin, it is helpful to report similar cases with immunohistochemical studies for future pathogenesis.

A preoperative clinical differential diagnosis includes metastasis, especially in old age, as well as myxoma, rhabdomyoma, fibroma, and leiomyosarcoma. Cardiac myxoma is commonly located within the left atrium and is composed usually of hypo-cellular bland stellate to ovoid reticulated cells in a myxoid background, which were not seen in our present case. Cardiac rhabdomyoma and fibroma are commonly detected in pediatric patients. Rhabdomyomas are usually composed of clear vacuolated cells with the presence of classical spider cells, while fibromas and benign neural tumors are composed of fascicles of interlacing spindle cell growth. They can be differentiated with the aid of S-100, which react strongly positive to nerve sheath tumors. Fibromas are usually seen in children under the age of one year and are composed of mostly bland fibroblastic proliferation in a collagenous background. Diffuse SMA, MSA, and H-caldesmon are strongly evident and support diagnoses of leiomyoma. Leiomyosarcoma is a malignant smooth muscle neoplasm which required two or more morphologic criteria for diagnosis: increase pleomorphic cytologic atypia, tumor necrosis, or mitoses more than 10/10 HPF [5]. Which was not present in our case.

## Conclusion

Primary intra-cardiac leiomyomas are rare benign mesenchymal tumors. All previous reported cases in the literature showed right ventricle predilection, which were proven histologically to be benign spindle cell lesions and were treated by surgical excision with no evidence of recurrence. Signaling abnormality in SFH can lead to congenital heart diseases. Thus, tumor pathogenesis theories are suggestive of SHF, which is a rich source for multipotent cellular progenitors. However, further studies are required to provide a valid tumorigenesis hypothesis. A clear embryogenic mechanism of action may improve proper understanding of cardiac tumors.

## Data Availability

Data are available on request to the corresponding author.
